# Queer voices in computational biology: the first ISCB LGBTQI+ Symposium

**DOI:** 10.1093/bioadv/vbae204

**Published:** 2025-02-06

**Authors:** R Gonzalo Parra, Mark N Wass, Seth Munholland, Mallory L Wiper, Diane Kovats, Lorena Pantano, Roland L Dunbrack

**Affiliations:** Life Sciences Department, Barcelona Supercomputing Center, Barcelona 08034, Spain; School of Biosciences, University of Kent, Canterbury CT2 7NZ, United Kingdom; International Society for Computational Biology (ISCB), Leesburg, Virginia, VA 20176, United States; International Society for Computational Biology (ISCB), Leesburg, Virginia, VA 20176, United States; International Society for Computational Biology (ISCB), Leesburg, Virginia, VA 20176, United States; Bioinformatics Core, Harvard T.H. Chan School of Public Health, Boston, MA 02115, United States; Institute for Cancer Research, Fox Chase Cancer Center, Philadelphia, PA 19111, United States

## Abstract

The first ISCB LGBTQI+ Symposium, held during Pride Month in 2024, marked a significant milestone for the International Society for Computational Biology (ISCB) community to promote diversity. This event aimed to provide a safe and supportive space for LGBTQI+ members of the society to share their experiences, address unique challenges in Bioinformatics and Computational Biology, and foster strategies for creating an equitable environment within ISCB. Through keynote presentations, short talks, and a roundtable discussion, participants explored topics such as minority stress, visibility, and the impact of role models. The symposium was rooted in a recognition of the historical and ongoing marginalization faced by LGBTQI+ individuals and sought to challenge systemic barriers while emphasizing the importance of community and representation. This article details the journey behind organizing the symposium, including overcoming the challenges of ensuring inclusivity and privacy, and highlights the profound impact of role models and collective action in advancing LGBTQI+ equity in science.

## 1 Introduction

According to various population studies, ∼10% of people self-identify as being part of the LGBTQI+ community, which includes Lesbian, Gay, Bisexual, Transgender, and other non-gender-conforming identities often referred to as non-binary or queer (Sell et al. 1995, Rahman et al. 2020). In recent years, the word “queer” has been reclaimed from its original pejorative meaning and adopted as an umbrella term that unifies all these identities. It is often used as a synonym for LGBTQI+, emphasizing that these categories are not static but often dynamic and exist on a continuum. Although each identity within this community faces specific challenges and advocates for their own needs, all of them share similar struggles in modern society. Historically, the different identities within this community have been subjected to various forms of discrimination related to their sexual orientation, gender identity, gender expression, or their phenotypic and behavioral characteristics.

The origins of homophobia and LGBTQI+ phobia involve a combination of cultural, religious, psychological, and social factors, with evidence that can be traced back to the Assyrian empire (that later influenced Judaism and Christianity) or the Greco-Roman period (Deyoung 2000, Fone 2001). Regardless of the reason, and though they have always been part of human societies, LGBTQI+ people have been historically persecuted and discriminated against. A pivotal point in western societies occurred during riots at the Stonewall Inn in New York City in 1969 where groups of LGBTQI+ people, led by Black transsexual women, confronted the police as a consequence of the constant abuses they were suffering. These riots sparked the movement for LGBTQI+ rights that have since expanded globally and that are annually commemorated at the end of June in Pride celebrations. Several milestones were achieved following the riots, such as removing homosexuality from the list of mental disorders in 1973 and the approval of same-sex marriage in the Netherlands in 2001 (currently legal in 36 countries).

Regardless of the many advances in the last decades, as of 2024, homosexuality remains criminalized in 64 countries, with punishment ranging from years of imprisonment to the death penalty (https://database.ilga.org/criminalisation-consensual-same-sex-sexual-acts). Even in those regions where LGBTQI+ identities are not criminalized (132 countries), stigma and discrimination are common. As stated by the United Nations, “*LGBTQI+ people are often discriminated against in the labor market, in schools and in hospitals, mistreated and disowned by their own families. They are singled out for physical attack—beaten, sexually assaulted, tortured and killed*” (https://www.un.org/en/fight-racism/vulnerable-groups/lgbtqi-plus). According to the minority stress model introduced by Meyer in (2003), LGBTQI+ people are subjected to different stressors that stem from the conflict between their identities and society’s dominant values. These can be classified into external stressors—such as discrimination, prejudice, and violence—that individuals experience because of their marginalized status and internal stressors that stem from the anticipation of discrimination, concealment of identity, and internalized LGBTQI+ phobia. It has been shown that suppression and concealment are related to adverse health outcomes. In contrast, expressing and disclosing traumatic events or characteristics of the self improve health by reducing anxiety and promoting assimilation of the revealed characteristics (Stiles 1995, Meyer 2003). Psychological studies have demonstrated the positive impact on self-esteem of affiliation with other similarly stigmatized people (Postmes and Branscombe 2002). The shared identity of members of stigmatized groups provides a basis for receiving social support and engaging in collective action to resist the perceived injustice of discrimination and prejudice (Haslam et al. 2005).

This article reports on the experiences behind the organization of the first ISCB LGBTQI+ Symposium held during the 2024 Pride Month. The event featured three keynotes and eight short talks. While some speakers offered testimonials about how their identities intersected with their professional development, others preferred to focus on strictly scientific aspects of their research. A highlight of the event was a lecture by Prof. Tom Waidzunas, who summarized various studies on LGBTQI+ populations, offering a statistical perspective that complemented the more personal narratives shared by other speakers. In addition, a roundtable discussion was held to explore various aspects of the experiences of LGBTQI+ individuals in STEM fields.

The aim of the symposium was to generate a safe forum where LGBTQI+ International Society for Computational Biology (ISCB) members could gather to discuss the challenges they face as professionals in the fields of bioinformatics and computational biology as a consequence of external and/or internal stressors, and to find strategies to alleviate such stress in order to create a more inclusive and welcoming atmosphere for the LGBTQI+ community within ISCB-related activities.

## 2 Precedents and overcoming the closet invisible barrier

By 2018, at the age of 30, R. Gonzalo Parra, who by then was serving as the ISCB Student Council (ISCB SC) vice chair, was experimenting with profound changes in his life as he internally worked out how to make it public within his social and professional circles that he recently started to experience homosexual relationships. At the time, Gonzalo was a postdoctoral researcher at the European Molecular Biology Laboratory (EMBL), where the institution’s policies to provide various forms of visibility and support to the LGBTQI+ community made him feel safe and protected. EMBL’s LGBTQI+ policies helped him to find the courage to come out publicly. By owning his queer identity, Gonzalo felt that he could help others who may be experiencing the same issues. This was especially important as he perceived that there were no LGBTQI+ role models to look up to in the field. In 2019, Gonzalo proposed a rainbow version of the logo for one of the ISCB SC’s flagship symposia to be included in promotional material as a sign of support to the LGBTQI+ community. Although the ISCB SC leadership was not against it, concerns about the potential negative backlash were raised, and the idea was dropped. Once the COVID-19 pandemic arrived and virtual events became widely adopted, Gonzalo thought of contacting a group of queer researchers to organize a virtual session about LGBTQI+ interests and its intersection with being a professional in the field of bioinformatics and computational biology. However, after contacting a few individuals, an unforeseen obstacle emerged: A fundamental difference between this event and other types of symposia is that in order to invite a person to speak on behalf of the LGBTQI+ community, we would first need to be completely sure that the individual is openly part of the community. Despite there being hints about someone being part of the community, it would not be respectful to address that person without having explicit evidence of them being out of the closet. Such an action could out that individual without their consent or misidentify them as part of the community based on cis-heteronormal stereotypes.

Gonzalo’s first approach to overcome this obstacle was to become vocal about LGBTQI+ topics on social media, especially on X (formally Twitter), which meant going through a new coming out, as exposing this dimension of his private life was still limited to a few people in the professional area. This second coming out is how Gonzalo got in contact with Prof. Roland L. Dunbrack Jr, a widely recognized computational biologist, who was also very open about being a gay man, with a long history of activism around LGBTQI+ issues. With this meeting, the perception Gonzalo had about the lack of LGBTQI+ role models in his field vanished. Role models existed; there was just no easy way to connect them with the younger members of the community. Thus, the necessity to create a forum to facilitate the networking among LGBTQI+ people in the fields of Bioinformatics and Computational Biology became more evident. Gonzalo and Roland agreed to collaborate to that end.

Finally, after becoming the Student Council’s representative on the ISCB Board of Directors in 2023, Gonzalo took the discussion to the board, where he asked for explicit institutional support for LGBTQI+ members of ISCB. The support for that call came in immediately as ISCB provided all the needed resources to organize the first ISCB LGBTQI+ Symposium in 2024. After some promotional material was designed, promotion of the event was made from the institutional social media accounts managed by ISCB, and a general call was made to recruit interested people to become part of the organizing committee. This was the solution to break the invisible closet barrier, as only people who were willing to be associated with the event would get in contact with the main organizers and not the other way around.

## 3 The event

Promotion started several months in advance on different social media platforms from ISCB, the ISCB SC, and from the organizers themselves. Everything was progressing smoothly until a series of coordinated negative comments began to appear, targeting posts about the event. These comments employed a variety of conservative, and occasionally religious, arguments. ISCB quickly addressed the comments and arguments, citing its ISCB Safe Policy. ISCB Safe was developed to maintain an environment that allows science and scientific careers to flourish through respectful, inclusive, and equitable treatment of others. The Society has a strong commitment to providing a safe place for its members and non-member participants alike. Thus, we used the ISCB Nucleus platform for the event to ensure a sense of safety and respect for presenters and attendees. ISCB technical support was present during the entire event, moderating the chat to avoid inappropriate behavior from attendees.

The event had an average of 30 attendees over the 5-h symposium with a peak of 43 attendees. We believe that the number of attendees may have been affected due to individuals potentially being hesitant to participate and have their names listed on the attendance record. This was needed for safety reasons but may have had an impact on attendance. All talks are publicly available through the ISCB YouTube channel https://youtube.com/playlist?list=PLmX8XnLr6zeH2-XKlYloEY3a6H5uFb160&si=UJzNoojJY10RX2XV

## 4 Roland L. Dunbrack Jr—from pink triangles to rainbow flags: 35 years in structural bioinformatics

Prof. Dunbrack talked about how he discovered that he was gay at around 10 years old, more or less at the same time he discovered his passion for math and science in general. He recalled that although during high school he had a math teacher who was gay, and who used to be seen around town with his partner, people would not really come out of the closet in general. Growing up, he had the idea but not the certainty that other people, men in particular, could be gay.

The situation turned out worse at the end of high school, in 1981, when a “rare type of cancer” appeared to be affecting homosexuals in New York and California, killing eight people in 2 years. This was the beginning of the HIV pandemic, when little was known about the disease, including its cause and transmission. Prof. Dunbrack started to internalize that being gay was somehow dangerous because he could get a deadly disease but also, more generally, because social stigma was compounded with fear of contagion. Therefore, he decided to rigidly stay inside of the closet for some time. This decision coincided with the time during his undergraduate studies when he started to work at the laboratory of Prof. Martin Karplus at Harvard and discovered his passion for protein research. Thanks to his undergraduate studies and research, in 1985 he received a Herchel Smith Fellowship to study at Cambridge University. He moved to England after college because he felt he needed to escape in order to better handle the process of accepting his sexuality.

Once in England, the pressure and stress of dealing with his sexuality, and the fear because of the HIV pandemic, started to take a serious toll on his mental health, with very dark thoughts crossing his mind. He mentioned that his life was saved, thanks to meeting a woman who was a graduate student in English literature in the same college. She was wearing a pin with an inverted pink triangle. The pink triangle was a sign to mark homosexuals and trans people at Nazi concentration camps, which later was positively re-appropriated as a symbol of lesbians and gays. For the first time, he had found somebody with whom he could openly talk about these feelings and the experience of being homosexual. She convinced him to attend an afternoon party at the college a month later, where he wore the same pink triangle pin for the first time as a way to come out to his social circle at the university. To his surprise, his friends reacted either warmly or nonchalantly.

After the party, when he returned home, he came out to a close friend, while still feeling ashamed about being gay and stressed about having just come out to dozens of people. Far from being shocked or negatively surprised, his friend convinced Roland to go out to the college bar and offered him company during that moment and helped him to overcome his feelings of shame. After this period, Roland and the friend who helped him come out founded a society of lesbian and gay graduate students in Cambridge from which a long path of activism began. He understood that LGBTQI+ friends and groups helped him to find his way out of the closet, and that it was important to be visible to others who were some steps behind in the process.

After some time, Roland came back to the USA and did his PhD at Harvard under the supervision of Prof. Karplus in the field of biophysics, where one of his first studies was about the conformation characteristics of prolines. This led him to publish his famous backbone-dependent rotamer library for proteins (Dunbrack Jr and Karplus 1993) and to develop a very successful career in the field. During his time at Harvard, he started another group for LGBTQI+ PhD students and continued to take part in other LGBTQI+ groups, proudly using the inverted pink triangle as a symbol to embrace diversity for himself and others.

After being a postdoctoral fellow at the University California, San Francisco (UCSF), Prof. Dunbrack is currently a Professor and research group leader at the Fox Chase Cancer Research Center in Pennsylvania, where he is focused both on contributing to our fight against cancer and also being a role model for the younger queer generations at his workplace and on social media. He points out that over his lifetime, the rainbow flag has replaced the pink triangle as a positive symbol of the LGBTQI+ movement. Even more so, the progress flag, now in common use, better reflects the diverse identities in our community.

## 5 Lorena Pantano—transforming shame into pride: my journey in bioinformatics

Lorena started to study biochemistry in Granada, Spain, in 2000. She mentioned that until that moment, she had not really taken too much time to think about her sexuality. It took a few years after starting college for Lorena to have a relationship with a woman. This relationship lasted a couple of years, but during that time, she kept it completely hidden from others in her social environment. Lorena recalls that after the first year or so, having to maintain a secret relationship became increasingly difficult. She also could not be open about the relationship, as being public about homosexuality was not an easy thing to do during that period in her small town in the south of Spain. As time passed, the difficulty of the secret relationship became too much, leading Lorena to break up with her partner as she approached her graduation in 2005.

After graduating, Lorena moved to Barcelona for her master’s studies in bioinformatics, after which she started a PhD in biomedicine in the field of non-coding RNA. Her PhD was very fruitful, leading her to deepen the understanding of the regulatory role of non-coding RNA, developing tools like SeqBuster (Pantano et al. 2010) and SeqCluster (Pantano et al. 2011), which are widely used. Lorena remembers that she was very focused on her career during her first years in Barcelona and did not feel the need to disclose to others about being a lesbian. This changed by the time she got to the middle of her PhD, in 2009, when she met the woman who eventually became her wife. Lorena described that this new relationship, which quickly became very important to her, in addition to living in Barcelona, far from her family, helped her to slowly come out to some close friends and co-workers. The story with her family was a little different. When they first discovered that she may have a girlfriend at college, there was a harsh discussion about this not being something good for her, but nothing more was said, and the topic was avoided until she finished her PhD. After her doctorate studies, Lorena became a postdoctoral researcher in the field of epigenetics and genomic alterations in 2011–4. Lorena had previously discussed with her partner the idea of moving to the USA to continue her career, but to do this, they would need to get married. When the decision to move across the ocean became more serious, she decided to properly discuss her coming out with her family, starting with her mother. Though she was already 30 years old and had accomplished much, Lorena recalls being very scared of letting her family down while also being troubled about these feelings. Her mother assimilated the idea well and decided she should be the one to communicate the news to Lorena’s father and brothers. The new reality took a while to sink in, but eventually it was accepted, and Lorena got married.

Lorena found and applied to an open position call at the Bioinformatics core in the Chan School of Public Health at Harvard. After the interview process and receiving the offer letter for the position, Lorena felt the need to disclose her sexuality to her recruiter to be sure that the environment at the workplace was going to be safe for her. Luckily, the answer was positive and allowed Lorena to accept the position, move to the USA, and further develop her career by taking part in different projects such as bcbio, bioconda, DEGReport, and Bioconductor. During the symposium, Lorena reflected on the intersectionality of being a lesbian and a woman and how this led her to also create the Boston Women in Bioinformatics group. From that time, every time she would become part of a new group, Lorena would try to make her sexuality obvious as soon as possible to normalize it in all her environments.

In 2019, she moved to the biotech sector, working for companies like eGenesis Tx, Arcella Tx, and NextRNA Tx. During that time, Lorena established sequencing platforms and genomics data dashboards internally to accelerate the path to clinical trials. Each of these moves, within the biotech world, required going through successive coming outs to ensure that the spaces were safe. Lorena understood with time that this was not only good for her, but would also pave the road for others, including a gay colleague who felt inspired to make the decision to come out in their work environment for the first time. In 2024, Lorena came back to the Chan Harvard School, where she is in charge of transferring new technologies from the biotech sector to the academic one and building platforms that will accelerate their applicability to treat human diseases. Lorena finished her presentation with a reflection that being a lesbian woman can be difficult sometimes for reasons such as receiving comments about her inadequacy from other people, having to correct others when they ask her about her husband instead of her wife, or experiencing different types of microaggressions, which, at the end of the day, result in her having to come out over and over again. However, this is something that she does with joy most of the time as she feels that exposing herself as a visible lesbian woman might support others in the same way visible people were good roles for her when she just started to accept herself.

## 6 Tom Waidzunas—LGBTQ+ STEM professionals: systemic inequalities and forms of resistance

The lectures by Prof. Dunbrack and Dr. Pantano were about personal stories about how two scientists from different generations and geographical locations, one identifying as a gay man and the other as a lesbian woman, went through life developing their professional careers while at the same time embracing their identities as part of the LGBTQI+ community. As bioinformaticians, we are aware that anecdotal examples do not represent populations as a whole, and therefore we wanted this event to be part of sharing a more data-driven assessment of how LGBTQI+ professionals feel and perform within the scientific ecosystem in STEM disciplines. For this, we invited Prof. Tom Waidzunas as a speaker. Tom is a Professor at Temple University in Philadelphia, whose research interests are at the intersections of sociology of sexuality, gender, science, technology, and social movement. Tom leads an NSF-funded project called the STEM inclusion study that aims to study the inclusion of STEM LGBTQI+ professionals in the context of their intersectionality with race/ethnicity, gender, and disability.

One of the first highlights from Prof. Waidzunas lecture was that research on the topic shows that LGBTQI+ people consistently experienced social exclusion and professional devaluation at work spaces. A study of biology colleges showed that while LGBTQI+ instructors were out to some of their colleagues, most were not out to their students due to fears of losing credibility, despite considering that it would be good for young LGBTQI+ students to have a role model.

Tom’s team conducted a survey with the participation of 25 324 people, out of which 1006 identified as LGBTQI+ and belonged to 21 STEM professional societies in the USA. In addition, the researchers performed 125 interviews, mostly comprising LGBTQI+, but also including some allies. Some of the results of this study were published in 2021 (Cech and Waidzunas 2021). LGBTQI+ respondents significantly reported having fewer career opportunities, fewer resources, and being less comfortable with whistleblowing inappropriate behavior from others. At the workplace, LGBTQI+ workers reported experiencing significantly more professional devaluation, being more socially excluded (e.g. not being invited to lunch or witnessing homophobic jokes or comments), and to have experienced different forms of harassment in comparison to non-LGBTQI+ participants. In the health dimension, LGBTQI+ professionals reported having significantly more minor health problems, increased levels of insomnia, higher stress levels, and showed more depression symptoms. At the same time, LGBTQI+ people had significantly more frequently thought about leaving STEM jobs or were actually planning to do so.

LGBTQI+ people face particular challenges when trying to find a job or integrating into the workplace. A study comprising different groups at NASA has shown that LGBTQI+ people have more difficulties working on short-term projects than on longer-term ones because shorter-term missions require constantly coming out to the new colleagues and gives less time to LGBTQI+ people to find strategies to integrate into their work teams (Cech and Waidzunas, 2022). LGBTQI+ workers spend extra mental energy to assess the levels of safety at their work spaces and become extremely cautious about their behavior and interactions with others, finding the need to come out as a measure to evaluate how much safety they find afterwards. LGBTQI+ workers and students make it a priority to find workplaces or institutions that already have LGBTQI+ associations in place or explicitly mention LGBTQI+ friendly policies to help narrow down their options.

A very important outcome from the NSF project was related to evaluating how the LGBTQI+ dimension intersects with other minority identities such as gender, race/ethnicity, and disabilities (Cech 2022). On the most privileged side of the spectrum, we can find heterosexual white men without disabilities while at the other extreme, we can find LGBTQI+ Black women with disabilities.

To end on a positive note, Tom mentioned that he and some colleagues are working on a book called “Out doing science: LGBTQI+ STEM professionals and inclusion in neoliberal times” where they recapitulate the emergence of various LGBTQI+ activist organizations. Since one of the first expressions of concerns within the American Association for the Advancement of Science (AAAS) (Escoffier et al. 1980) was the negative effects of homophobia on LGBTQI+ scientists and the direct or indirect damage it represented to the scientific system as a whole, multiple associations have emerged to fight for a more equitable, inclusive, and diverse scientific community. In what Waidzunas calls the “education era,” spanning from 1983 to 2005, activists needed to argue that homophobia in STEM corrupts scientific knowledge. There was a need for gay and lesbian perspectives in STEM, but coming out as LGBTQI+ could be a reason for being fired. Later on in the “professional development era (2005–present),” inclusion is considered fundamental to prevent loss of talent. It has been recognized that diversity promotes innovation, that people can be more efficient and loyal if they can be their full selves at work, and coming out as LGBTQI+ became institutionally easier as these identities are valued as part of the innovation and productivity assets under many equity diversity and inclusion (EDI) perspectives.

## 7 Discussion and future directions

Scientific research, like any other human endeavor, inherently involves a social dimension, where researchers operate within a rich social, cultural, and historical context. As much as science is often presented as a pristine, objective activity, it is not possible for scientists to completely dissociate their professional and private lives. In similar ways as it happens with women or non-white people who face different forms of discrimination and barriers to progress, the LGBTQI+ community has its own obstacles and vicissitudes. Some of them are in the form of external stressors, while others come from internal processes that stem from the intrinsic trauma from growing up being different in a cis heteronormative society that did not accompany their emotional and psychological development in a supporting and healthy way. Moreover, even as adults, LGBTQI+ people often need to adjust, tone down, or hide parts of who they are to avoid harassment while in public, either in professional or extra-professional settings.

The testimonies from Prof. Roland L. Dunbrack Jr and Dr. Lorena Pantano represent, unfortunately, the stories of a majority of LGBTQI+ people, who experience fear and shame as teenagers or in their early adulthood while they discover that they are different from the rest of their peers. It shows how many individuals from the LGBTQI+ community feel the need to move away from home to avoid conflicts with their families and walk through the process of self-discovery and self-acceptance in solitude until they feel ready to speak out to others for the first time. Their stories also reflect how, by coming out, many of the negative issues start to get solved as they approach others with similar experiences, often starting to participate in various forms of LGBTQI+ activism. Another common issue relates to the fact that LGBTQI+ people need to come out every time they change their social environment to be sure they can inhabit a safe space. This limits their possibilities for professional development, as many places, although excellent, are not a realistic option because of their lack of LGBTQI+-oriented policies.

The lecture by Prof. Waidzunas shows that the struggles experienced by Prof. Dunbrack and Dr. Pantano are not simply anecdotal but statistically prevalent in the LGBTQI+ community. Being different in their sexuality and gender dimensions represents a heavy mental burden for individuals that holds them back from professional progress because of poor mental health outcomes and being targets of different forms of harassment by others.

Several of the organizers of the first ISCB LGBTQI+ Symposium expressed similar experiences, mentioning that in most cases, they navigated them without institutional support, isolated from others in similar situations. The aim behind this event was to generate a forum where LGBTQI+ members of ISCB could talk to each other about these issues. A place where they could feel cathartic about the struggles and obstacles they perceive and experience, and to design strategies to support each other across their professional development. This virtual event represented a pilot experience of contact, followed up by a small in-person activity at ISMB in Montreal in July 2024, where some of the attendees met to continue the discussion and plan future activities, perhaps with an in-person edition of the symposium to become a dedicated session at future ISMBs.

Being different from the societal norm is challenging, but progress has been made. In 1954, Alan Turing, the father of theoretical computer science, died 2 years after being chemically castrated due to his homosexuality. Only 68 years later, Prof. Svante Paabo, openly bisexual, was awarded the Physiology and Medicine Nobel Prize for his work on the study of ancient human genetics. This was the first time that the award was given to an LGBTQI+ individual who was out of the closet. There were previous awardees who were suspected of being part of the community but who had lived their entire lives in secret as a way to protect themselves from the negative consequences of it. Today, being part of the LGBTQI+ community does not represent a serious threat for most people living in western societies. A positive sign of it, as shown by Dr. Gaoxiang Zhou, an educator in the USA who presented at the symposium, might be how the proportion of students who identify themselves as part of the LGBTQI+ community has steadily increased since 2010. However, various forms of discrimination persist, and therefore awareness strategies need improvement to integrate members of the LGBTQI+ community in a healthy and harmonious way in the scientific system.

The organizers of the first ISCB LGBTQI+ Symposium deeply thank the ISCB leadership who have fully supported this initiative from the first moment it was proposed. We believe that the support of allies is crucial to advance in the eradication of LGBTQI+ phobia and other types of hate and discrimination from society. Nobody needs to be alone while being different, and sometimes it just takes a small, kind action to support a person who is struggling to help them get out of darkness. We firmly believe that initiatives like the ISCB LGBTQI+ symposium will help members of the LGBTQI+ community to feel welcome in a supportive environment, as well as to help younger LGBTQI+ people to have role models to look up to, and go through the process of self-acceptance in the company of others. Together, we will continue to make ISCB a more equitable, inclusive, and diverse community.
